# Effect of Dexmedetomidine Hydrochloride on perioperative inflammation and postoperative lung infection in patients with spinal tuberculosis

**DOI:** 10.12669/pjms.37.2.2383

**Published:** 2021

**Authors:** Yong-hui Liu, Ye Zhao, Xiang-yang Wang, Hong-xun Cui

**Affiliations:** 1 Yong-hui Liu, Department of Orthopedics, Henan University of Chinese Medicine, Zhengzhou, Henan 450000, P.R. China, Department of Orthopedics, Orthopedic Hospital of Henan Province, Luoyang, Henan 471000, P.R. China; 2 Ye Zhao, Department of Orthopedics, Henan University of Chinese Medicine, Zhengzhou, Henan 450000, P.R. China, Department of Orthopedics, Orthopedic Hospital of Henan Province, Luoyang, Henan 471000, P.R. China; 3 Xiang-yang Wang, Department of Orthopedics, Henan University of Chinese Medicine, Zhengzhou, Henan 450000, P.R. China, Department of Orthopedics, Orthopedic Hospital of Henan Province, Luoyang, Henan 471000, P.R. China; 4 Hong-xun Cui, Department of Orthopedics, Orthopedic Hospital of Henan Province, Luoyang, Henan 471000, P.R. China

**Keywords:** Dexmedetomidine hydrochloride, Inflammatory factor, Perioperative period, Spinal tuberculosis

## Abstract

**Objective:**

To explore the effect of dexmedetomidine hydrochloride on perioperative inflammation and postoperative lung infection in patients with spinal tuberculosis.

**Method:**

A double-blind control observation was conducted in spinal tuberculosis patients with the use of general anesthesia during the operation. A total of 171 spinal tuberculosis patients who received endotracheal intubation for general anesthesia in Henan University of Chinese Medicine from January 2017 to April 2019 were included. The concentration changes in serum TNF-α and IL-6 were recorded at one hour, six hour and one day after the operation. The incidence of postoperative pulmonary complications of patients were also evaluated.

**Results:**

The results showed that in the experimental group compared with the control group, serum TNF-α and IL-6 concentrations one hour, six hour and one day after the operation were significantly lower (P<0.05). The rate of postoperative pulmonary complications was lower in the experimental group than that in the control group (P<0.05).

**Conclusion:**

Dexmedetomidine hydrochloride has obvious anti-inflammatory effects and can reduce the incidence of pulmonary complications after surgery.

## INTRODUCTION

Alpha2 adrenoceptor stimulation can resist stress and inflammatory factors, and its potential clinical application value is being studied by people.^1^-^3^ Dexmedetomidine hydrochloride (Dex) represents a new generation of α2 adrenoceptor stimulants. It excites α2 adrenoceptors and produces effects of calming, analgesia and sympatholytic effects via high selectivity. Dex can stabilize hemodynamics, reduce sympathetic nerve activity and result in anti-inflammatory actions via the high selectivity of the agonist in the locus coeruleus in the brainstem and of peripheral adrenergic receptors.^4^,^5^ In addition, it can avoid the adverse reactions from clonidine α1 receptor excitement, with strong predictability in terms of pharmacokinetics. Dex is considered a pure α2 adrenoceptor stimulant by clinicians.^6^ Spinal tuberculosis is the most common form of musculoskeletal tuberculosis and surgery is the main treatment method for spinal tuberculosis.^7^-^9^ Present study observed and analyzed the effects of Dex on concentration changes in tumor necrosis factor (TNF-α), interleukin (IL-6), and pulmonary inflammation in spinal tuberculosis patients during the perioperative period with the hope of providing value for clinical application.

## METHODS

A total of 171 spinal tuberculosis patients who received endotracheal intubation for general anesthesia in Henan University of Chinese Medicine from January 2017 to April 2019 were included and allocated to into the experimental Group-A (93 patients) and the control Group-B (78 patients) according to the number table method for double-blind contrast observation. The American Society of Anesthesiologists (ASA) grading was grades I-III.

### Inclusion criteria:

1. Patients with spinal tuberculosis surgery, patients aged 18-60 years old, and patients with endotracheal intubation for general anesthesia.
2. No obvious abnormality of liver, kidney or lung functions and no history of chronic pulmonary inflammation.
3. No obvious electrolyte disturbance or infection, no bradycardia (heart rate >55 times ·min-1), and no atrioventricular block before the surgery.
4. No use of α2 adrenoceptor stimulant sin the past 3 months. The differences in age, body mass and operation time in both groups were not statistically significant (P<0.05), as shown in Table-I.

**Table-I:** Comparison of the general data.

*Group*	*No.*	*Age/year*	*Body mass/kg*	*Operation/min*
A	93	46.9±16.7	57.2±13.6	200.0±15.9
B	78	47.4±15.5	53.9±14.9	203.9±10.9
t	-	-0.202	1.513	-1.83
P value	-	0.841	0.132	0.068

### Ethical approval

The study was approved by the Institutional Ethics Committee of Henan University of Chinese Medicine on February 19, 2020, and written informed consent was obtained from all participants.

### Anesthesia method

Anesthesia induction: for Group-B, midazolam (0.03~0.05 mg·kg^-1^), sufentanil (0.2 ~0.5 μg·kg^-1^), etomidate (0.15 ~ 0.20 mg·kg^-1^), and cisatracurium besylate (0.15~0.25 mg·kg^-1^) were intravenously injected. Then, mechanical ventilation was conducted after endotracheal intubation under a visual laryngoscope. In Group-A, before anesthesia induction, Dex (0.5 μg·kg^-1^) was pumped intravenously for 15 minutes. Then, the dose and speed were adjusted to 0.4 μg·kg^-1^·h^-1^ for continuous infusion until the main surgical operations were completed. Maintenance of anesthesia: 10~20 μg sufentanil was intravenously injected in an interrupted manner. Propofol (2~5 mg·kg^-1^·h^-1^) was intravenously injected with a micropump. Then, 1.0% ~3.0% sevoflurane and remifentanil (0.1 ~ 0.3 μg·kg^-1^·min^-1^) were inhaled. The bispectral index was maintained at 40~50 during the operation, and cisatracurium besylate was intravenously injected in an interrupted manner to maintain muscle relaxation. SpO2 was maintained above 97%. The partial pressure of carbon dioxide in end-expiratory gas was maintained at 3.5~ 4.0 kPa. The central body temperature was maintained within the normal range (36.5~37.7°C).

### Observation index

The concentration changes in serum TNF-α and IL-6 were recorded at different time points. An enzyme-linked immunosorbent assay (ELISA) was used to determine serum TNF-α and IL-6 concentrations after entering the operating room and before anesthesia (T_1_), one hour after the operation started (T_2_), six hour after the operation ended (T_3_) and one day after the operation ended (T_4_) (5 ml of fasting venous blood was extracted and centrifuged in a tube without the addition of anticoagulation, and the upper serum was removed). Pulmonary complications after the operation were recorded. Diagnostic criteria of pulmonary complications:

1. Pulmonary infection
2. ***Hypoxemia:*** partial pressure of arterial oxygen (PaO2) <7.98 kPa, oxygenation index < 53.067 kPa, or SpO2 <90%.
3. ***Pulmonary Atelectasis:*** poor oxygenation and low lung breath sounds on the affected side or chest X-ray showing that lung tissue permeability is low accompanied by the mediastinum; the septum transversum of the hilus pulmonis on the affected side moves to the atelectasis area; and compensatory hyperventilation of the healthy lung.

### Statistical method

SPSS20 software was used for statistical analysis. Normally distributed measurement data were expressed as the mean ± standard deviation (x±s). Independent sample t-test was adopted for intergroup mean comparisons, and the repeated measurement of data variance was adopted for intragroup mean comparisons. Enumeration data were compared with the χ^2^ test. P<0.05 indicates that the difference was statistically significant.

## RESULTS

The concentration differences of serum TNF-α and IL-6 in both group at T_1_ had no statistical significance. Compared with T_1_, at T2, T3 and T4, serum TNF-α and IL-6 levels rose obviously (P<0.05). Compared with Group-B, in Group-A, at T2 ~T4, serum TNF-α and IL-6 levels were significantly lower (P<0.05), as shown in Table-II.

**Table-II:** Comparison of TNF-α and IL-6 levels at each time point (x ± s).

*IL-6*	*T1*	*T2*	*T3*	*T4*
A	39.94±3.99	56.42±4.09	281.42±37.92	186.42±27.36
B	40.84±3.22	81.37±7.26	337.36±40.13	275.69±30.98
t	-1.602	-28.236	-9.356	-20.004
P value	0.111	<0.001	<0.001	<0.001

*TNF-α*	*T1*	*T2*	*T3*	*T4*

A	30.98±3.28	60.86±7.98	185.52±22.12	150.21±18.02
B	30.43±2.75	92.73±9.54	220.02±27.31	184.73±21.56
t	1.172	-23.790	-9.127	-11.406
P value	0.242	<0.001	<0.001	<0.001

Intragroup comparison at T_1_, ^* 1^P<0.05.

The incidence rate of postoperative pulmonary complications in Group-A was obviously lower than that in Group-B, and the difference was statistically significant (P<0.05), as shown in Table-III.

**Table-III:** Comparison of postoperative pulmonary complications

*Group*	*No.*	*Pulmonary infection*		*Pulmonary atelectasis*		*Hypoxemia*		*Incidence rate/%*

		*n*	*%*	*n*	*%*	*n*	*%*	
A	93	0	0	1	1.08	1	1.08	2.15
B	78	3	3.85	6	7.69	6	7.69	19.23
*X^2^*	-	3.641		4.731		4.731		-
*P* value	-	0.058		0.030		0.030		-

## DISCUSSION

TNF-α and IL-6 levels are increased in patients with spinal tuberculosis.^10^,^11^ The time of operation is long, and the complications of hypoxemia, pulmonary atelectasis and pulmonary infection may occur. Postoperative pulmonary complications (PPCs) include pulmonary atelectasis, pneumonia, bronchitis, and respiratory insufficiency.^12^ The incidence rates of inflammation, pulmonary infection or infection of other tissues are high, which affects postoperative recovery.^13^-^15^ Dexmedetomidine is a potent, highly selective α-2 adrenoceptor agonist, with sedative, analgesic, anxiolytic and sympatholytic, which can reduce abnormally increased blood pressure and heart rate under stress states.^16^,^17^ Taniguchi et al.^18^ verified that Dex could lower the death rate of endotoxemia rats and the effect of the inflammatory response, and serum TNF-α and IL-6 levels were clearly lower. Moreover, Dex also has organ protection effects and can prevent apoptosis 19 Ref pl. In the process of the inflammatory response, the inflammatory promoter TNF-α and the strongest inflammatory mediator IL-6 among pro-inflammatory cytokines have the strongest sensitivity and can quickly reflect the severity of the inflammatory response caused by surgical stress.^20^,^21^ In this study, compared with group B, serum TNF-α and IL-6 levels at T2 ~T4 decreased significantly in group A. It has been reported that during renal ischemia-reperfusion, Dex alleviates the inflammatory response and eases tissue damage by regulating the level of inflammatory factors.^22^

The incidence rate of postoperative pulmonary complications in Group-A was obviously lower than that in Group-B (P<0.05), indicating that Dex can lower the reactivity of inflammatory cells and inhibit proinflammatory factor release and granulocyte aggregation in the lung, thus alleviating pulmonary function damage. It seems that Dex has an obvious anti-inflammatory effect and can reduce postoperative pulmonary complications. This study also showed that the dose of sufentanil and other drugs during the operation was obviously lower than that of Group-B, indicating that Dex has high affinity and can be used together with other analgesia drugs and intravenous general anesthesia drugs at the same time to generate good drug superposition effects and to significantly reduce the anesthetic dose.

It shows that dexmedetomidine hydrochloride has important clinical significance in patients undergoing spinal tuberculosis surgery. However, the research on the clinical value of dexmedetomidine hydrochloride in this study is limited to patients undergoing spinal tuberculosis surgery. As for its application value in other diseases, further research is needed in the future.

### Limitations of the study

Only typical inflammatory factors were selected. Later studies need to look at more inflammatory factors and study the interaction relationship.

## CONCLUSION

In conclusion, Dex may have anti-inflammatory actions and can reduce the release of TNF-α and IL-6 in spinal tuberculosis patients during the perioperative period. This can inhibit the inflammatory response in these patients. Dex can decreases the incidence rate of postoperative pulmonary complications in patients of caries spine.

### Authors’ Contributions:

**YL and YZ** designed this study and prepared this manuscript, they are responsible and accountable for the accuracy or integrity of the work.

**HC** collected and analyzed clinical data.

**XW** significantly revised this manuscript.
